# The quality of care for adults with epilepsy: an initial glimpse using the QUIET measure

**DOI:** 10.1186/1472-6963-11-1

**Published:** 2011-01-03

**Authors:** Mary Jo Pugh, Dan R Berlowitz, Jaya K Rao, Gabriel Shapiro, Ruzan Avetisyan, Amresh Hanchate, Kelli Jarrett, Jeffrey Tabares, Lewis E Kazis

**Affiliations:** 1 South Texas Veterans Health Care System, VERDICT REAP, (7400 Merton Minter), San Antonio, TX, (78229), USA; 2 University of Texas Health Science Center at San Antonio, Department of Epidemiology and Biostatistics, (7703 Floyd Curl Drive), San Antonio, TX, (78229) USA; 3 Center for Health Quality, Outcomes and Economic Research (CHQOER), Veterans Administration Medical Center, (200 Springs Road), Bedford, MA, (01730), USA; 4 Boston University School of Public Health, Health Policy and Management, (715 Albany St, Talbot Building), Boston, MA, (02118), USA; 5 University of North Carolina, Eshelman School of Pharmacy, (Kerr Hall, Room 2202) Chapel Hill, NC, (27599), USA; 6 Hôpital Sainte-Justine, Université de Montréal, (3175, Chemin de la Côte-Sainte-Catherine), Montreal, Quebec, (H3T 1C5), Canada; 7 Boston Veterans Administration Medical Center, Center for Organization, Leadership, and Management Research, (150 South Huntington Ave.), Jamaica Plain, MA, (02130), USA; 8 Center for the Assessment of Pharmaceutical Practices (CAPP) and Pharmaceutical Assessment Management and Policy (PAMP), Department of Health Policy and Management, Boston University School of Public Health, (715 Albany St, Talbot Building), Boston, MA, (02118), USA

## Abstract

**Background:**

We examined the quality of adult epilepsy care using the *Quality Indicators in Epilepsy Treatment (QUIET) *measure, and variations in quality based on the source of epilepsy care.

**Methods:**

We identified 311 individuals with epilepsy diagnosis between 2004 and 2007 in a tertiary medical center in New England. We abstracted medical charts to identify the extent to which participants received quality indicator (QI) concordant care for individual QI's and the proportion of recommended care processes completed for different aspects of epilepsy care over a two year period. Finally, we compared the proportion of recommended care processes completed for those receiving care only in primary care, neurology clinics, or care shared between primary care and neurology providers.

**Results:**

The mean proportion of concordant care by indicator was 55.6 (standard deviation = 31.5). Of the 1985 possible care processes, 877 (44.2%) were performed; care specific to women had the lowest concordance (37% vs. 42% [first seizure evaluation], 44% [initial epilepsy treatment], 45% [chronic care]). Individuals receiving shared care had more aspects of QI concordant care performed than did those receiving neurology care for initial treatment (53% vs. 43%; X^2 ^= 9.0; p = 0.01) and chronic epilepsy care (55% vs. 42%; X^2 ^= 30.2; p < 0.001).

**Conclusions:**

Similar to most other chronic diseases, less than half of recommended care processes were performed. Further investigation is needed to understand whether a shared-care model enhances quality of care, and if so, how it leads to improvements in quality.

## Background

While existing quality indicators have focused on a number of highly prevalent chronic conditions (e.g., diabetes, hypertension) they do not address the quality of care for less prevalent, but serious conditions, such as epilepsy. Epilepsy care presents complexity in the sense that providers must balance seizure control, adverse drug effects, and complicated issues associated with epilepsy itself (e.g. mood disorders [1-3]) while also being mindful of consequences related to long-term treatment with antiepileptic drugs (e.g. bone health [4-6]). Thus, it is important to begin examining the quality of care provided to patients with epilepsy using quality measures and identifying gaps in quality of care. The United Kingdom has begun this process [7] due to the availability of not only clinical guidelines for care for patients with epilepsy[8,9], but also quality indicators from the Quality and Outcomes Framework [7]. While no comprehensive national guidelines for care of patients with epilepsy exist in the United States, the development of the QUality Indicators for Epilepsy Treatment in adults (QUIET) allows us to begin to examine the quality of epilepsy care in the United States.

The purpose of this study is to describe the quality of care received by adults with epilepsy in a major medical center in a Northeastern US city using the QUIET indicators-quality indicators developed as part of a larger study funded by the Centers for Disease Control and Prevention (CDC; Additional file 1)-and to assess the quality of epilepsy care in primary care and general neurology settings. Similar to other countries, in the US a substantial number of patients continue to receive their epilepsy care solely within the context of primary care (55% in one study) [10]. Studies of quality of care for other chronic diseases have found better quality of care among patients receiving care from medical sub-specialists or within a shared care context [11-13]. Thus, we examine the extent to which variations exist in quality of care among patients who received epilepsy care only within primary care, only within neurology subspecialty care, and within both neurology and primary care (shared). Based on findings from previous studies, we hypothesize that patients with epilepsy are more likely to receive high quality care when they receive specialty care exclusively or have epilepsy care shared by both primary care and neurology specialty care [14-16].

## Methods

### Data

Data from the electronic medical record of a single medical center in the northeastern United States were used in this study to identify patients with epilepsy and assess the extent to which recommended processes of care were performed. The electronic medical record includes templates for certain aspects of care such as vital signs, medications, and lab tests. However, as progress notes are not disease specific or used for examining quality of care at the institution, they are primarily free text, which allows substantial variation in documentation of the care provided. Data were acquired from the demographic information, diagnosis codes, patient problem list, pharmacy, laboratory, inpatient, and outpatient components of the medical record. These data were entered into a specially designed chart abstraction form, and entered into a spreadsheet and exported to SPSS (Version 17.0, Chicago, IL) for subsequent analysis. This study was approved by the Institutional Review Board of the Medical Center.

### Sample

Eligible participants received care at the medical center, were at least 18 years of age, able to speak English and, due to an additional component of the larger study, were able to complete a telephone interview. Probable epilepsy patients were identified by searching the electronic medical record system for all patients who had at least one ICD-9 code of 345.x or 780.39, or mention of the terms "epilepsy" or "seizure disorder" in the medical record problem list between 2004 and 2007. In order to assure there would be adequate data to assess the quality of epilepsy care we further required that individuals have two or more visits to the primary care or neurology clinics, or one visit to the primary care or neurology clinic and at least one hospitalization [17] between 2004 and 2007.

Individuals identified as eligible for the study based on diagnosis of epilepsy and who were confirmed to have epilepsy by their primary care provider or neurologist and received a letter from their physician informing them about the study and inviting their participation. Chart abstractions examining the quality of epilepsy care were completed by trained clinical chart abstractors only for those participants who contacted the study team after receiving the letter and provided written consent to participate in the study.

### Quality Indicators

QUIET quality indicators were developed using the RAND appropriateness method-a process that integrates a systemic literature review with an expert consensus process to identify items that are considered appropriate and feasible measures of quality healthcare. Since existing evidence based guidelines from international sources existed, we adapted some items from those existing guidelines (e.g,, National Institute for Clinical Excellence [NICE] and Scottish Intercollegiate Guidelines Network [SIGN]), for use in the US healthcare setting [8,9]. Other items were developed using systematic literature review. A panel of 10 epilepsy experts and 1 primary care provider completed three rounds of ratings to identify items for which there was consensus regarding appropriateness, feasibility and necessity. Additional file 1 shows the QI's that were rated appropriate and feasible indicators of epilepsy care quality, and further identifies QI's that were adapted from the NICE or SIGN guidelines, and items which are parallel to measures included in the Quality and Outcomes Framework measure used in the United Kingdom [18]. Additional detail about this process is provided elsewhere [19].

Because of the large number of individual evidence-based QI's during the development of the quality measures, we categorized the 22 QI's into four aspects of epilepsy care: First Seizure Assessment (3 QI's), Initial Epilepsy Treatment (7 QI's), Chronic Epilepsy Care (9 QI's), and Aspects of Care Unique to Women (3 QI's). Additional file 1 shows specific QI's for each aspect of care.

### Operational definitions of quality indicators

Prior to chart abstraction, the research team consisting of physicians, nurses and health services researchers with expertise in conducting chart abstractions created operational definitions that identified specific data elements that would be used to score each QI. These definitions were then used to determine if applicable conditions were present for measuring each QI (i.e. the IF portion of the quality indicator), and then whether the process of care defined by the quality indicator was provided (i.e. the THEN portion). For example: IF the patient is diagnosed with a seizure disorder/epilepsy and started on therapy (denominator) THEN the patient should be treated with monotherapy (numerator). Thus, there was variation in the denominator by QI. While some QI's focused on a single visit (at the time of the first seizure), most QI's examined the construct in question over time. For instance, a number of QI's recommended that a certain type of care be provided on a yearly basis (e.g. yearly depression screening). For these, we assessed visits within the designated time frame. Thus, our analysis is at the patient level for each QI, and the total number of individuals who met criteria for each QI was unique.

A specially designed chart abstraction instrument was developed, and data regarding processes of care provided was collected and entered using Microsoft Access. Research assistants with clinical background were specifically trained to perform chart reviews (Additional file 2). The final chart abstraction instrument was subjected to multiple layers of review by the research team. Initially, 25 charts were reviewed by two raters to assess concordance. Comparison of ratings for the quality indicators in those charts found that raters agreed 76% of the time. After finalization of the instrument, a second review of 25 charts conducted by independent raters found that there was agreement on 86% of ratings.

Initial assessment of concordance between QI's and care provided used an all-or- nothing approach: if all aspects of care were performed, the patient received a positive score for that indicator unless the indicator specifically stated that one of several aspects of care would fulfill the requirement (e.g. QI15; see Additional file 1). However, we also examined the proportion of recommended processes of care completed for each of the four aspects of epilepsy care described above.

### Patient Characteristics

Demographic characteristics including age, sex, race/ethnicity, marital status, and education were abstracted from the electronic medical record. Epilepsy history (new-onset vs. chronic care), type of seizure (if documented), and medication information was ascertained based on longitudinal review of medical records and intake history for patients new to the medical center. Finally, the presence of continued seizures was identified by documentation of seizures in the medical record during the study period.

### Setting of Epilepsy Care

Epilepsy care was identified by review of outpatient progress notes that described epilepsy and epilepsy care. For each epilepsy-focused medical encounter (encounter in which progress notes mentioned epilepsy) within the healthcare system, the type of provider was defined as being a primary care provider (general internist, family practitioner, or nurse practitioner), neurologist, or other specialty care. Individuals who received care only within primary care or neurology settings were classified as such. Those receiving epilepsy care in both settings were classified as receiving shared care.

### Analysis

We first describe the variation in concordance among individual QI's (i.e., the concordance between the recommended and actual care received), followed by examination of the proportion of patients who received the care outlined for each aspect of epilepsy care (First Seizure, Initial Epilepsy Treatment, Chronic Care, Aspects of Care Unique to Women; Additional file 1 shows indicators included in each aspect of care). This analysis was conducted at the patient level, but included all recommended aspects of care for which each patient was eligible based on the individual indicators in each aspect of epilepsy care. This process is described in more detail below. Finally, we examine receipt of care by the setting of epilepsy care received using the chi square statistic. Haberman's adjusted residual statistic was used to identify significant cells within the chi-square analysis [20]. SPSS^® ^version 17 (Chicago, IL, USA) was used to conduct data analysis.

## Results

The sample for this study consisted of 311 individuals. Table 1 shows demographics for the sample overall and by source of care. Overall, approximately 58% were women, 62% were between 18 and 49 years of age; the sample was racially diverse with similar numbers of whites and African Americans. Over half had some college or a college degree. With regard to epilepsy, approximately 21% had new-onset epilepsy (within the past two years) and the median number of antiepileptic drugs (AEDs) prescribed at the last visit for this sample was 1 (mean = 1.41, SD = .90). Forty seven percent continued to have seizures during the study period, and 66% had no change in AED during the course of the study. Examination of demographic characteristics (Table 1) by source of care found that African Americans were more likely to be in shared care and primary care groups and less likely to be in the neurology group than expected (X^2 ^= 18.8, df = 2; p < 0.01). Individuals with continued seizures were less likely to be the primary care group and more likely to receive shared care than expected by chance (X^2 ^= 21.5, df = 2; p < 0.01). Individuals within the primary care group were less likely to have a medication change over the course of the study than expected by chance (X^2 ^= 9.6, df = 2; p = 0.01).

**Table 1:** Characteristics of Study Participants

	All	Neurology Only N (%)	Shared care N (%)	Primary Care Only N (%)
Total	311	203 (65.27)	77 (24.76)	31 (9.97)
**Sex**				
Female	181 (58.20)	114 (56.16)	50 (64.94)	17 (54.84)
Male	130 (41.80)	89 (43.84)	27 (35.06)	14 (45.16)
**Age**				
18-49	194 (62.38)	131 (64.53)	46 (59.74)	17 (54.84)
50-64	80 (25.72)	46 (22.66)	24 (31.17)	10 (32.26)
65+	37 (11.90)	26 (12.81)	7 (9.09)	4 (12.90)
**Race/Ethnicity**				
White	147 (47.27)	112 (55.17)*	27 (35.06)*	8 (25.81)*
African American	133 (42.77)	70 (34.48)*	42 (54.55)*	21 (67.74)*
Other	31 (9.97)	21 (10.34)	8 (10.39)	2 (6.45)
**Education**				
Less than high school	50 (16.29)	28 (13.86)	13 (17.33)	9 (30.00)
High school graduate	91 (29.64)	63 (31.19)	23 (30.67)	5 (16.67)
Some college	93 (30.29)	57 (28.22)	23 (30.67)	13 (43.33)
College graduate	73 (23.78)	54 (26.73)	16 (21.33)	3 (10.00)
**New-Onset Epilepsy**	65 (20.90)	45 (21.17)	16 (20.78)	4 (12.90)
**Number of Epilepsy Medications** **(Last clinic visit)**				
0	32 (10.29)	19 (9.36)	7 (9.09)	6 (19.35)
1	166 (53.38)	109 (53.69)	41 (53.25)	16 (51.61)
2	76 (24.44)	51 (25.12)	19 (24.68)	6 (19.35)
3 or more	37 (11.90)	24 (11.83)	10 (12.99)	3 (9.68)
**No Antiepileptic Drug Change**	207 (65.56)	133 (65.52)	46 (59.74)	28 (90.32)
**Continued seizures**	147 (47.27)	90 (44.33)*	51 (66.23)	6 (19.35)*
Education is self-reported				

*p < 0.01

There was substantial variation in concordance between recommended care and actual care for individual QI's (Table 2). Excluding indicators with a total denominator of less than 25 (to ensure precision of analyses) concordance ranged from 2% for QI16 to 99% for QI's 15 and 18. The mean proportion of concordant care by indicator was 54.0 (standard deviation = 28.0); the median was 59.26.

**Table 2:** Proportion of Patients Receiving QI Concordant Care by Setting of Care

Quality Indicator	All N = 311	Neurology only N = 203	Shared care N = 77	Primary Care Only N = 31
**Evaluation of First Seizure**				
**QI 1.**	22/65 = 33.85%	11/45 = 24.44%	10/16 = 62.5%	1/4 = 25%
**QI 2.**	25/65 = 38.46%	18/45 = 40%	7/16 = 43.75%	0/4 = 0%
**QI 3.**	17/21 = 80.95%	12/16 = 75%	3/3 = 100%	2/2 = 100%
**Initial Treatment of Epilepsy**				
**QI 4.**	29/65 = 44.62%	21/45 = 46.67%	8/16 = 50%	0/4 = 0%
**QI 5.**	10/65 = 15.38%	5/45 = 11.11%	4/16 = 25%	1/4 = 25%
**QI 6.**	34/46 = 73.91%	23/31 = 74.19%	10/11 = 90.91%	1/4 = 25%
**QI 7.**	21/23 = 91.3%	15/15 = 100%	6/6 = 100%	0/2 = 0%
**QI 8.**	17/64 = 26.56%	12/44 = 27.27%	5/16 = 31.25%	0/4 = 0%
**QI 9.**	NA	NA	NA	NA
**QI 11.**	20/31 = 64.52%	12/21 = 57.14%	7/9 = 77.78%	1/1 = 100%
**Follow-up/Chronic Disease Care**				
**QI 14.**	76/272 = 27.94%	53/176 = 30.11%	23/66 = 34.85%	0/30 = 0%
**QI 15.**	145/147 = 98.64%	90/90 = 100%	51/51 = 100%	4/6 = 66.67%
**QI 16**	6/311 = 1.93%	2/203 = 0.99%	4/77 = 5.19%	0/31 = 0%
**QI 17.**	16/27 = 59.26%	12/19 = 63.16%	4/8 = 50%	
**QI 18.**	69/70 = 98.57%	43/43 = 100%	26/27 = 96.3%	
**QI 19.**	82/132 = 62.12%	50/83 = 60.24%	30/46 = 65.22%	2/3 = 66.67%
**QI 20.**	142/311 = 45.66%	72/203 = 35.47%	53/77 = 68.83%	17/31 = 54.84%
**QI 21.**	88/125 = 70.4%	53/73 = 72.6%	22/34 = 64.71%	13/18 = 72.22%
**QI 22.**	11/14 = 78.57%	6/7 = 85.71%	5/7 = 71.43%	
**Aspects of Care Specific to Women**				
**QI 23. **I	38/111 = 34.23%	24/73 = 32.88%	13/29 = 44.83%	1/9 = 11.11%
**QI 24.**	1/5 = 20%	0/2 = 0%	1/3 = 33.33%	
**QI 25.**	8/12 = 66.67%	3/6 = 50%	5/5 = 100%	

QI number is based on the original QI's reported in Pugh MJ, Berlowitz DR, Montouris G *et al*. What constitutes high quality of care for adults with epilepsy? Neurology 2007; 69(21):2020-7. QI were not deemed appropriate and necessary (QI 10, 12, 13) are not presented in this paper.

Table 2 shows the proportion of patients who received QI concordant care for each QI overall and by the setting of epilepsy care. The only QI's where sufficient primary care patients were represented for the purposes of analysis were in chronic epilepsy care: QI 14, 15 16, 20, 21, and 23. For these aspects of chronic epilepsy care we found no statistically significant differences in the extent to which patients received referral for treatment after a positive depression screen (QI 21; X^2 ^= 0.73, df = 2; p = 0.7), or folate supplementation for women of childbearing age (QI 23: X^2 ^= 3.6, df = 2; p = 0.2) by the setting of epilepsy care. Haberman's Adjusted Residual analysis of statistically significant analyses indicated that patients who received primary care were less likely than expected to have documentation of approximate seizure count since their last visit (QI 14; X^2 ^= 13.6, df = 2; p = 0.001) or interventions performed in light of continued seizures documentation of patient education (QI 15: X^2 ^= 47.6, df = 2; p < 0.001). Individuals who received care in neurology and primary care settings were less likely to receive depression screening than those who received shared care (QI 20 X^2 ^= 6.2, df = 2; 26.21; p < 0.001).

Table 3 shows the proportion of QI concordant care among the four aspects of epilepsy care (first seizure evaluation, initial epilepsy treatment, chronic epilepsy care, and aspects of care unique to women) overall and by setting of epilepsy care. The number reported in each column represents the proportion of all possible care processes that were performed among the care processes outlined by the QI for those who met QI inclusion criteria. For evaluation of a first seizure, of the 65 individuals who received a first seizure evaluation there were 151 possible opportunities for care among individuals who met inclusion criteria regarding QI's for first seizure assessment (QI 1-3). Of those 151 opportunities for QI concordant care, 64 were completed (42.38%). Among the 65 individuals who met criteria for QI's examining initial treatment of epilepsy, there were 297 opportunities for recommended care. QI concordant care was provided for 131 (44.11%) of those opportunities. Among the 311 individuals who met criteria for many of the chronic epilepsy care QI's, there were 1,409 opportunities for recommended care; 45.07% of the time QI concordant care was provided. Among the 111 women of child-bearing age included in the study there were 128 opportunities for recommended care; 36.72% of the time QI concordant care was provided. These data indicate that overall, less than half of all possible QI identified care processes (877/1985; 44.2%) were completed in this sample. The lowest concordance between recommended care and actual care was for aspects of care unique to women.

**Table 3:** Proportion of all Possible Opportunities Taken for Quality Epilepsy Care by Setting of Epilepsy Care

Aspect of Epilepsy Care	N***	All	Neurology Only	Shared care	Primary Care Only
First seizure assessment^$^	63	64/151 = 42.38%	41/106 = 38.70%	20/35 = 57.14%	3/10 = 30.00%
Initial epilepsy treatment	63	131/297 = 44.11%	88/203 = 43.35%	40/75 = 53.33%	**3/19 = 15.79%**
Chronic epilepsy care	302	635/1409 = 45.07%	381/897 = 42.47%	**218/393 = 55.47%**	36/119 = 30.25%
Aspects of care unique to women^$^	108	47/128 = 36.72%	27/81 = 33.33%	19/37 = 51.35%	1/10 = 10.00%

^$ ^Insufficient N for Primary Care Only; unable to compare.

* p < 0.05

**p < .01

***N that could potentially qualify for inclusion in analysis if all inclusion criteria for a specific quality indicator are met.

The numerator in each column represents the number of possible care processes that were performed; the denominator represents the number of possible care processes. For specific numbers of patients, and specific indicators included in each aspect of epilepsy care please refer to Table 2.

Further examination of quality within each aspect of epilepsy care suggests that there were also significant differences by the setting in which care was received. Our examination of differences between settings of care was restricted to neurology vs. shared care for First Seizure Assessment and Aspects of Care Unique to Women due to low numbers of patients receiving Primary Care Only. For First Seizure Assessment, we found a trend approaching significance with individuals receiving shared care having more aspects of QI concordant care performed than did those receiving care in a neurology setting (Table 3; X^2 ^= 3.7; p = 0.06). For Initial Epilepsy Treatment concordance by setting of care varied from 16% for those receiving primary care only to 53% for those receiving shared care; individuals receiving primary care were significantly less likely to receive QI concordant care than expected (X^2 ^= 9.0; p = 0.01). Concordance for QI's included in Chronic Epilepsy Care ranged from 30% for those receiving primary care only to 55% for those receiving shared care. Individuals receiving shared care were more likely to receive QI concordant care than those receiving neurology or primary care (X^2 ^= 30.2; p < 0.001). For Aspects of Care Unique to Women, there was a trend approaching significance, with individuals receiving shared care being more likely to receive QI concordant care than those receiving neurology care (X^2 ^= 3.5; p = 0.06).

## Discussion

This study used data from electronic medical records to assess the extent to which patients with epilepsy receive processes of epilepsy care identified as QI's designed for use in primary and general neurology care. We were able to reliably assess the extent to which recommended care was documented in patient records. However findings for several indicators where fewer than 5% of the sample received recommended aspects of care suggest additional evaluation of those indicators or data sources is needed.

Consistent with studies examining QI concordant care in other chronic diseases, less than half of all possible care processes were completed [21]. There was wide variation in the extent to which all recommended processes of care were provided, with Aspects of Care Unique to Women having the lowest rates of concordance.

Our data provide limited support for our hypothesis that individuals receiving shared care have better quality of care than individuals receiving only primary care or only neurology subspecialty care. The only QI's for which there were significant differences between patients receiving neurology care and those shared care was QI20 which suggests that persons with epilepsy should receive an annual depression assessment. Our data do not indicate who conducted the depression assessment. It is possible that the neurologist was more likely to attend to these items immediately for a patient that would be seen only once for referral, than the more complex patients with competing demands who were seen continuously in neurology settings [22]. The more complex patients who were seen exclusively in neurology settings likely had acute issues associated with their seizures that required immediate attention, leaving little or no time at the end of an office visit to address chronic disease management issues. Alternatively, these chronic disease management issues may have also been addressed by the neurologist but not documented due to the intensive documentation required to address more acute seizure care.

Further examination of the aggregate measures where there is more power to detect differences between individuals receiving shared vs. primary or neurology care only, our analysis revealed that there was a significant difference between shared care and neurology only care only for chronic disease management.

Shared care has been examined in a variety of contexts with mixed results. A number of studies have found no difference in quality for patients in shared care compared to those in specialty care exclusively [23,24]. Rosendal and colleagues found that individuals with hip fracture and receiving shared care had more home care after discharge, and lower scores on the short version of the Sickness Impact Profile indicating improved recovery [25]. Pugh and colleagues found that patients with diabetes were more likely to receive guideline concordant diabetes medications if they received care from both primary and specialty care compared to primary care or specialty care exclusively [26].

Our analysis suggests that, for epilepsy, QI's associated with types of care that are more technical in nature (e.g., QI 2, 7, 15, 18) tended to have higher rates of concordance, while those associated with discussion, patient education, and chronic disease management (e.g., QI 5, 8, 16) tended to have lower rates of concordance. It is possible that these aspects of patient care that are more interpersonally oriented were actually performed, but not documented. However, documentation of these aspects of care is in and of itself an indicator of quality. Because several of these interpersonally oriented items are ones included in the UK's Quality and Outcomes framework, there is broad international consensus that they are critical components of the quality of epilepsy care (e.g., particularly documenting seizure frequency and reviewing medication management). Accordingly the QUIET indicators could be used to develop electronic templates for use in documenting care for patients with epilepsy in organizations with electronic medical records (or a paper template for settings with paper records). Such a template would facilitate documentation of these important processes of care when they occur, and guide providers who do not commonly provide care for patients with epilepsy.

Beyond our hypothesis, we found that chronic disease management issues specific to women tended to have lower rates of concordance than other aspects of care. Again, it is possible that women were taking over-the-counter medications or vitamins with the recommended dose of folate and did not need a prescription. However, the drug-drug interaction between lamotrigine and oral contraceptives was not addressed in four of five eligible patients. Because the numbers of women meeting criteria was low for QI 24 women's issues should be highlighted as a concern not only for clinicians to address, but also as a concern requiring additional quality assessment.

Finally, our findings suggest that issues recently highlighted in the literature-bone health and mood disorders [27,28] -are being addressed for many patients with epilepsy. Approximately 62% of individuals on AEDs for two or more years received testing for Vitamin D or a DXA scan. With regard to mood disorders, 46% received recommended screening. Of those with evidence of anxiety or depression, 71% received treatment with medications or a referral to a mental health practitioner.

These findings must be interpreted in light of several limitations. First, this study was in a single medical center in which study participants appeared to have higher rates of uncontrolled seizures than estimates from the literature [29]. The complexity of these patients may be such that competing demands for physician time make addressing less acute issues more difficult, so these findings may not reflect the quality of care provided for less complicated patients. Attempts to adjust these results for disease severity, however, did not change results since there were no significant differences in QI concordant care for those with and without continued seizures. This limited sample, from a single geographic location provides a point of reference for future studies from other geographic regions and multi-site studies with power to detect geographic variations in care that may exist.

Related to this single-site study is the fact that the total number of patients (N = 311) was relatively small, particularly after stratifying patients into groups of neurology care, shared care and primary care. The primary care group was particularly small for all groups except chronic epilepsy care. Thus, findings for the primary care group must be interpreted with caution.

Next, quality of care was assessed primarily using the electronic medical record. As described above, it is possible that interpersonal aspects of care were addressed but not documented. A number of aspects of care that are more interpersonal in nature may be better assessed using patient reported survey measures. Future studies examining the quality of epilepsy care using a broad measure such as the QUIET indicators may benefit from an approach that utilizes not only medical chart abstraction, but a targeted survey that provides a second assessment of patient reported receipt of care which allows one to triangulate data to assess the care received by patients. Moreover, such a survey would benefit by adding quality of life measures, and other potential outcomes that may be linked to higher quality care such as employment, relationship status, and education.

We further found that scoring compound QI's (e.g., 1, 8 and 16) in an all or nothing fashion may lead to loss of useful information. Adaptation and/or disaggregation of specific aspects of these QI's may be needed in future research on the quality of epilepsy care. However, our assessment of broad areas of epilepsy care (Table 3) examined the proportion of possible care processes that were completed. Even in this analysis where credit was given for completing even one aspect of care for complex QI's, fewer than half of all possible processes of care were completed, suggesting room for improvement in all aspects of epilepsy care.

Finally, because these QI's were designed for use in primary or general neurology care settings, it is possible that some very important aspects of care may not be as clearly articulated. For instance we indicated numerous points where referral to a higher level of specialty care is recommended. Due to the structure of care in the US, we did not specifically identify a point in time where a patient should be referred to a tertiary epilepsy center for comprehensive evaluation. This limitation should be addressed by future versions of this measure.

## Conclusion

This study provides a snapshot of the quality of care provided to adults with epilepsy in one metropolitan healthcare system. While results are only from a single hospital, these findings suggest that assessment of quality using most of the QUIET QI's is feasible, and that care for epilepsy can in large part be reliably measured using medical chart abstraction. This tool will allow identification of gaps in quality for epilepsy care within other healthcare systems, and eventually it can be used to improve the care provided for adults with epilepsy. However additional evaluation of the QUIET measure using patient surveys and development of QI's for use in specialty care is also needed.

## Competing interests

Dr. Pugh reports no disclosures

Dr. Berlowitz reports no disclosures

Mr. Shapiro reports no disclosures

Dr. Montouris reports no disclosures

Dr. Avetisyan reports no disclosures

Dr. Hanchate reports no disclosures

Ms. Jarrett reports no disclosures

Mr. Tabares reports no disclosures

Dr. Kazis reports receiving research funding for other projects from Eli Lilly and Company, Bristol Meyers Squibb, Sanofi-Aventis, Genzyme, Boehringer- Ingelheim, Amgen, and Janssen Pharmaceuticals

## Authors' contributions

MJP contributed to acquisition of funding, concept and design of the study, interpretation of data, and preparation of manuscript. DRB contributed to acquisition of funding, concept and design of the study, analysis and interpretation of data, and preparation of manuscript. GS MJP contributed to data acquisition, analysis of data, and preparation of manuscript. GDM MJP contributed to interpretation of data, and preparation of manuscript. RA contributed to data acquisition and preparation of manuscript. AH contributed to analysis and interpretation of data and preparation of manuscript. KJ MJP contributed to data acquisition and preparation of manuscript. JT contributed to analysis of data and preparation of manuscript. LEK contributed to acquisition of funding, concept and design of the study, interpretation of data, and preparation of manuscript. All authors read and approved the final manuscript.

## Pre-publication history

The pre-publication history for this paper can be accessed here:

http://www.biomedcentral.com/1472-6963/11/1/prepub

## Supplementary Material

Additional file 1 **Quality in epilepsy treatment in adults (QUIET) indicators**. This file includes the specific quality indicators included in the QUIET measure. Click here for file

Additional file 2 **CDC epilepsy quality of care study: patients' medical record review/abstraction form**. This file includes the chart abstraction form used to collect data on quality of care from patients' electronic medical record. Click here for file
