# Supplementary material for: The quality of care for adults with epilepsy: an initial glimpse using the QUIET measure
Source: BMC Health Serv Res. 2011 Jan 3;11:1. doi: 10.1186/1472-6963-11-1 (PMC3024216; doi:10.1186/1472-6963-11-1)
Supplement: Additional file 1 — Quality in epilepsy treatment in adults (QUIET) indicators. This file includes the specific quality indicators included in the QUIET measure. [file 1472-6963-11-1-S1.DOC]

Additional File 1

|  | **Quality in Epilepsy Treatment in Adults (QUIET) Indicators**  **Evaluation of First Seizure**  **^*QI 1.** In the initial clinical evaluation of a first seizure, the patient should receive:  - detailed seizure history (events before, during, and after)  - review for predisposing conditions (i.e., stroke, head trauma, drugs/alcohol)  - physical and neurological examination  - labs (e.g., screening laboratory testing for routine medical assessment)  AND if there is no indication of provocation  - an order for EEG  - an order for neuroimaging (MRI preferred) or rationale for not ordering  OR Referral to higher level of epilepsy specialty care |
| --- | --- |
|  | **^*QI 2.** At the time of initial seizure evaluation, the patient should receive information on driving restrictions, safety and injury prevention. |
|  | **^*QI 3.** IF the individual with a first unprovoked seizure has any of the following:  - a neurological deficit  - an EEG which shows unequivocal epileptiform activity  - the individual and/or their caregivers consider the risk of having a further seizure more detrimental than a trial of AED treatment (e.g. employment or driving issues)  - brain imaging shows a structural abnormality  - Age over 60 years  THEN benefits and risks of AED treatment should be described and AED treatment offered, or reasons for not prescribing should be documented. |
| **Initial Treatment of Epilepsy** | |
| **^*QI 4.** IF a patient is thought to have a diagnosis of epilepsy THEN the diagnosis should include a best estimation of seizure types. | |
| **^*QI 5.** IF the patient meets the criteria for epilepsy diagnosis (generally two unprovoked seizures)  THEN AED treatment should be discussed with the patient and caregivers and offered. | |
| **^*QI 6.** IF the patient is diagnosed with a seizure disorder/epilepsy and started on therapy THEN the patient should be treated with monotherapy. | |
| **^*QI 7.** IF a patient is a woman of childbearing potential THEN referral to a neurologist or an epilepsy specialist is indicated. | |
| **^*QI 8.** During the visit at which a patient is diagnosed with a seizure disorder/epilepsy, the patient should receive information on:  -Driving restrictions, safety and injury prevention  -Diagnosis and treatment options including the importance of taking AEDs as directed  -Triggers and other lifestyle factors that may affect seizure control (e.g. sleep deprivation, alcohol/ drug use)  -Contraception and family planning | |
| **QI 9.** IF a newly diagnosed person with epilepsy is over the age of 60 and is not currently on any AED therapy, THEN use of enzyme inducing AEDs (phenobarbital, phenytoin, carbamazepine) should not be started unless at least two other AEDs have been unsuccessful in stopping seizures or have intolerable adverse effects. | |
| **^*QI 11.** IF a person newly diagnosed with epilepsy is taking medications for other disorders, THEN the physician should minimize the risk of interactions between the newly prescribed AED and concomitant medications. | |

Appendix A continued

| **Follow-up/ Chronic Disease Care** |
| --- |
| **^#^QI 14.** WHEN a patient with epilepsy receives follow-up care, THEN an estimate of the number of seizures since the  last visit and an assessment of drug side-effects should be documented. |
| ***QI 15.** IF the patient continues to have seizures after initiating treatment THEN interventions should be performed.  Options include:  - Compliance assessment/ enhancement  - Monitor AED blood levels  - Increase AED dose  - Change AED  - Patient education re: lifestyle modification  - Referral to higher level of epilepsy care |
| **^#^*QI 16.** Patients with epilepsy should receive an annual review of information including topics such as:  - Chronic effects of epilepsy and its treatment including drug side-effects, drug-drug interactions, effect on bone health  - Contraception, family planning, and how pregnancy and menopause may affect seizures (EVIDENCE GRADE C)  - Screening for mood disorders.  - Triggers and lifestyle issues that may affect seizures  - Impact of epilepsy on other chronic and acute diseases  - Driving and safety issues |
| **^*QI 17.** IF the patient reports unacceptable side-effects from AED monotherapy, THEN an alternative AED should be started (with carefully planned crossover). |
| ***QI 18.** IF use of at least two different AED monotherapies has not resulted in seizure freedom, THEN referral for more highly specialized epilepsy care is indicated. |
| **QI 19.** IF the patient is on AEDs for 2 or more years THEN providers should assess bone health. |
| **QI 20.** A person with epilepsy should receive screening for depression at least once each year. |
| **^QI 21.** IF a person with epilepsy is found to have evidence of a mood disorder (e.g., depression, anxiety), THEN s/he should receive treatment or a referral for mental health care. |
| **^*QI 22.** IF a person with epilepsy is well controlled THEN s/he should have an annual review of adverse effects of drugs and self-management issues. |

Appendix A continued

| **Aspects of Care Specific to Women** |
| --- |
| **QI 23.** IF a woman with epilepsy is of childbearing potential THEN she should receive daily supplemental folate at a dose of at least 400 mcg. |
| **QI 24.** IF a woman with epilepsy is of childbearing potential and receives oral contraceptives in conjunction with an enzyme inducing AED, THEN decreased effectiveness of oral contraception should be addressed. (higher doses of the oral contraceptive, alternative birth control methods, or change AED). |
| **QI 25.** Prenatal care for a woman with epilepsy should be co-managed by a neurologist and an obstetrician with experience in high risk pregnancy to assure that issues related to the impact of epilepsy and its treatment on the pregnancy are addressed. |

**^ Adapted from Scottish Intercollegiate Guidelines Network Guidelines**

*** Adapted from National Institute for Clinical Excellence Guidelines**

**^#^ Parallel to Quality and Outcomes Framework**
