# Supplementary material for: The quality of care for adults with epilepsy: an initial glimpse using the QUIET measure
Source: BMC Health Serv Res. 2011 Jan 3;11:1. doi: 10.1186/1472-6963-11-1 (PMC3024216; doi:10.1186/1472-6963-11-1)
Supplement: Additional file 2 — CDC epilepsy quality of care study: patients' medical record review/abstraction form. This file includes the chart abstraction form used to collect data on quality of care from patients' electronic medical record. [file 1472-6963-11-1-S2.DOC]

**CDC Epilepsy QUALITY OF CARE STUDY**

***Patient’s Medical Record Review/Abstraction Form***

***Instructions for chart abstractor: Visit specific information should be reviewed for last two years, from telephone interview date.***

| **PATIENT DEMOGRAPHICS**  **Instructions for chart abstractor**  **Items 1-9:** *sign-in and click “Reg” tab (located on the top most row, 4^th^ from left) see “Patient” tab and view Personal box (on the top left side)*  **Items 10-11***: click the “Chart” tab (located on the most rows, second from left) then “Documents” then “All” or “Visits”.*  **Item12***: click the “Chart” tab (located on the top most row, second from left) then “Documents” (5th row, far right) then “All” or “Visits” then view HPI.* | | | | | | | | | | | | | |
| --- | --- | --- | --- | --- | --- | --- | --- | --- | --- | --- | --- | --- | --- |
| 1 | | Date of birth? | | MM/DD/YY | | | | | | NO DATA | | | |
|  | | ***1.1*** *60 or over?* | | YES | | | | | | NO | | | |
|  | | ***1.2***  *18-45 years of age?* | | YES | | | | | | NO | | | |
| 2 | | Sex | | MALE | | | | | | FEMALE | | | |
| 3 | | Patient Zip code | |  | | | | | | | | | |
| 4 | | Employment | | YES | | | | NO | | | | NO DATA | |
| 5 | | Primary Care Provider? | | YES | | | | NO | | | | NO DATA | |
|  | | ***5.1*** *Primary Care Provider Code* | |  | | | | | | | | | |
| 6 | | Ethnicity | | *Drop-down menu* | | | | | | | | | |
| 7a | | Language code | | *Drop-down menu* | | | | | | | | | |
| 7 | | Race | | *Drop-down menu* | | | | | | | | | |
| 8 | | Marital Status | | *Drop-down menu* | | | | | | | | | |
| 9 | | Does patient have primary medical insurance? | | YES | | | | NO | | | | NO DATA | |
|  | | 9.1 If Yes, specify Primary insurance/ payer | | *Drop-down menu* | | | | | | | | | |
|  | | 9.2 If No or No data, specify Secondary or other insurance, whatever is recent.  *Instruction: Click the “Reg” tab, go to “Patient” section and look for Secondary or Other Insurance in the “Insurance and Employment” box. If there is no data here, go to “Insurance” section; scroll down to the last entry to select the insurance with the most recent date.* | | *Drop-down menu* | | | | | | | | | |
| **10** | | **What is the number of specific visits made to BMC during the last 2 years?** | |  | | | | | | | | | |
|  | | (a) Number of visits PCP | |  | | | | | | | | | |
|  | | (b) Number of visits to Neurologist or Epileptologist*?* | |  | | | | | | | | | |
|  | | (c) Number of visits to Emergency Room | |  | | | | | | | | | |
|  | | (d) Number of hospitalizations | |  | | | | | | | | | |
|  | | (e) Number of specialty visits other than Neurology | |  | | | | | | | | | |
| 11 | | Has patient been hospitalized during the last year? | | YES | | | | NO | | | | NO DATA | |
|  | | ***11.1*** *How many times? (# of inpatient hospitalizations)* | |  | | | | | | | | | |
|  | |  | |  | | | | |  | | |  | |
| **CASE/DIAGNOSIS IDENTIFICATION**  **Instructions for chart abstractor**  **Items 13-15:** *: click the “Chart” tab (located on the top most row, second from left) then “Documents” (5^th^ row, far right) then “All” or “Visits” then view problem list and physician notes.* | | | | | | | | | | | | | |
| 13 | | New Epilepsy Case  *Note: Where [12/31/04] is marked in sub-items for 13-15, the date 2 years before the phone interview will be substituted.* | | YES | | | | | | NO | | | |
|  | | ***13.1*** *First epilepsy / seizure-related ICD-9 code assigned on or after [12/31/04] in problem list or physician notes, whichever comes first* | | YES | | | | | NO | | | NO DATA | |
|  | | ***13.2*** *First documentation of epilepsy/seizures/seizure disorder on or after [12/31/04], in problem list or physician notes, whichever comes first* | | YES | | | | | NO | | | NO DATA | |
| 14 | | Chronic Epilepsy Case | | YES | | | | | | NO | | | |
|  | | ***14.1*** *First epilepsy / seizure-related ICD-9 code assigned before [12/31/04] in problem list or physician notes, whichever comes first* | | YES | | | | | NO | | | NO DATA | |
|  | | ***OR***  ***14.2*** *First documentation of epilepsy/seizures/seizure disorder before [12/31/04] in problem list or physician notes, whichever comes first* | | YES | | | | | NO | | | NO DATA | |
| 15 | | Chronic but new to BMC | | YES | | | | | | NO | | | |
|  | | If 13 is Yes, | | YES | | | | | | NO | | | |
|  | | ***AND***  ***15.1*** *Patient is registered at BMC on or after [12/31/04]* | | YES | | | | | NO | | | NO DATA | |
|  | | ***AND***  ***15.2*** *Any mention in physician note that patient had Epilepsy or seizure disorder or Seizures before [12/31/04]* | | YES | | | | | NO | | | NO DATA | |
| **Not Visit Specific Questions**  **EPILEPSY DAIGNOSIS AND MANAGEMENT**  **Instructions for chart abstractor: Please go as far as possible. Please do not restrict to any time limits**  **Items16-20:** *click the “Chart” tab (located on the top most row, second from left) then “Documents” (5^th^ row, far right) then “All” or “Visits” then view Physician/RN’s notes and “Hospital Documents”, Emergency reports,Transcripts. Also view problems list.* | | | | | | | | | | | | | |
| 12 | | Family History of Epilepsy? | | YES | | | | | NO | | | NO DATA | |
| 16 | | Any diagnosis of epilepsy found in medical record?  Note- Diagnosis of epilepsy includes epilepsy disorder/ seizure disorder  If any of 13, 14 or 15 is yes- this should be automatically yes. | | YES | | | | | NO | | | NO DATA | |
|  | | 16.1 Is there any documentation by a physician that the patient had two or more “unprovoked” seizures?  ***Instructions:***  *This pertains to any occurrence of seizure(s) during the last year which meets our definition of active epilepsy.* | | YES | | | | | NO | | |  | |
| 17 | | Any documentation indicating that patient was diagnosed prior to enrollment at BMC | | YES | | | | | NO | | | NO DATA | |
|  | | ***17.1*** *How old was patient at diagnosis?* | |  | | | | | | | | | |
|  | | ***17.2*** *What is the date when the patient was diagnosed?* | |  | | | | | | | | | |
|  | | ***17.3*** *How long ago was diagnosis made?* | |  | | | | | | | | | |
|  | | ***17.4*** *What is the date of patient’s enrollment at BMC?* | |  | | | | | | | | | |
| 18 | | ***Instruction:***  *All questions in the section 18 and 18.x, except 18.6, are relevant to NEW epilepsy cases. We cannot answer to these questions for chronic cases, as for them we*   - *either don’t know when and how and by who the first AED was prescribed (because Logician goes only as far as 2000), or* - *the first diagnosis was made more than two years ago, and the information does not contribute to the objectives of our study (two year period).* | |  | | | | | | | | | |
| 18 | | Associated with epilepsy/seizure disorder diagnosis, was an AED prescribed? | | YES | | | | | NO | | | NO DATA | |
|  | | *Instructions for 18.1-18.5. If Q.18 is Yes, answer 18.1-18.5. Please look at the record when diagnosis was made or the first time AED appeared in patient’s records).* | |  | | | | |  | | |  | |
|  | | ***18.1*** *Was the first AED prescribed monotherapy or combination therapy?* | | YES | | | | | NO | | | NO DATA | |
|  | | ***18.2*** *If yes, please capture the date of documentation associated with the FIRST AED?* | | MM/DD/YY_____________ | | | | | | | | | |
|  | | ***18.3*** *If no AED was prescribed, is there evidence that physician documented a reason* | | YES | | | | | NO | | | NO DATA | |
|  | | ***18.4*** *Did provider explain benefits and/or risks of AED treatment?* | | YES | | | | | NO | | | NO DATA | |
|  | | ***18.5*** *Review list and select all AEDs prescribed for the date captured above.* | | *Drop-down menu* | | | | | | | | | |
|  | | **18.6 Documentation that Patient is on AED for two or more years** | | YES | | | | | | NO | | | |
| 38 | | What ICD-9 codes are captured in the problem list? (complete list) | |  | | | | | |  | | | |
|  | | 38.1 ICD-9 code | |  | | | | | |  | | | |
|  | | 38.2 Date | |  | | | | | |  | | | |
| **MISCELLANEOUS QUESTIONS** | | | | | | | | | | | | | |
| 19 | | Is there any evidence that the patient is seen for epilepsy by providers outside of BMC? | | YES | | | | | NO | | | NODATA | |
|  | | ***19.1*** *If “YES” What type of provider did the patient see?(provide list)* | | Neurology/Epileptologist/PCP/others | | | | | | | | | |
|  | | ***19.2*** *Was the patient referred to BMC by an outside physician?* | | YES | | | | | NO | | | NO DATA | |
| 20 | | Is the patient seeing a psychiatrist/counselor/social worker/psychologist?(both outside and within BMC) | | YES | | | | | NO | | | NO DATA | |
| **VISIT SPECIFIC QUESTIONS**  **Instructions for chart abstractor**  **Items21-39:**   1. Click the “Chart” tab, then “Documents” (5th row, far right) then “All” or “Visits” then view Physician/Nurse’s notes, Transcripts 2. DOCUMENT all the dates of the PCP and Neurology visits for last 2 years, starting from the earliest one. 3. REVIEW ONLY Epilepsy /Seizure disorder or Seizure related Neurology, PCP, and other specialty visits. Please also collect data from visits where Mood disorder, Oral Contraceptives, Bone health issues, and AED side-effects like thrombocytopenia, hyperplasia, hair loss, tremor and drowsiness, and others are discussed. 4. PCP visits where epilepsy is mentioned but not discussed should be entered as non-epilepsy-related. 5. Please capture the dates starting from the earliest visit to the recent visit***.*** | | | | | | | | | | | | | |
| 21 | DATE OF VISIT (MM/DD/YY) | | |  | | | | | | | | | |
|  | ***21.1*** *Indicate the type of visit?*  ***Instructions for abstractor***   1. PCP visit= any of PCP clinic, Adult clinic, Family Medicine, General Internal Medicine, Women's clinic, and Geriatrics. 2. PCP visits where epilepsy is mentioned but not discussed should be entered as non-epilepsy-related. 3. Review Nurse Visits (NP or RN visits) if those are documented as PCP visits. The same rules apply as in other PCP visits. 4. Note: there are specific codes for Neurologists, PCP doctors and PCP Nurses. 5. For PCP visits of women, select the checkbox, if the main reason of the visit is Women’s issues (including pregnancy, contraception, post menopausal issues, etc) 6. Do not review Office Procedures, Office transcripts or Telephone messages if those are not related to an Office visit. You should consider and review all these, if those are documented on the same day as the office visit and related/ supplement office visit note. | | | *Drop-down menu:*   - *Epilepsy-related neurology visit* - *Epilepsy-related PCP visit Main reason for visit is Women’s issue: Yes/ No* - *Epilepsy-related visit – other specialty* - *Non-epilepsy-related neurology visit* - *Non-epilepsy-related PCP visit Main reason for visit is Women’s issue/ Ob/Gyn problems: Yes/ No* - *Non-epilepsy-related specialty visit* - *Other* | | | | | | | | | |
| 22 | CODE OF PHYSICIAN | | |  | | | | | | | | | |
| 23 | DIAGNOSIS OF EPILEPSY/seizures/seizure disorder (based on physician notes) | | | YES | | | | | | NO | | | |
|  | 23.1 Enter diagnosis from physician notes –  Instructions for abstractor – Capture this from Impression or from list of diagnosis in physician notes) | | | Free Text | | | | | | | | | |
| 24 | DIAGNOSIS OF EPILEPSY BASED ON ICD-9 CODE in this visit( Instructions- Capture from physician notes) (defined as epilepsy-related ONLY) | | | YES | | | | | | NO | | | |
|  | ***24.1*** *Specify the epilepsy-related ICD-9 code in this visit* | | | *Drop-down menu* | | | | | | | | | |
| 25 | For this office visit, were any the following exams/tests done or specific results found? | | |  | | | | | | | | | |
|  | - 1. *Physical examination*   *Instructions for abstractors- For PCP physical exam contributes to Ht,Wt,BP,RR,Temp; For neurology visit- it constitutes Ht & Wt* | | | YES | | | | | NO | | | NO DATA | |
|  | - 1. *Neurological examination*   *Instructions for abstractor- If physician notes mentions examination- unchanged, assume it has been done* | | | YES | | | | | NO | | | NO DATA | |
|  | ***25.21*** *Neurological Deficit*  *Instructions for abstractor- Physician notes must clearly state “neurological deficit”* | | | YES | | | | | NO | | | NO DATA | |
|  | ***25.3*** *Labs (e.g. screening laboratory testing for routine medical assessment like CBC, Lipid Profile, HB, ) LIST (We need a list of Lab)* | | | YES | | | | | NO | | | NO DATA | |
|  | - 1. *EEG ordered or done* | | | YES | | | | | NO | | | NO DATA | |
|  | - 1. *Unequivocal Epileptiform Activity* | | | YES | | | | | NO | | | NO DATA | |
|  | ***25.5*** *Brain MRI ordered or done* | | | YES | | | | | NO | | | NO DATA | |
|  | ***25.51*** *Structural abnormality detected by MRI* | | | YES | | | | | NO | | | NO DATA | |
|  | ***25.6*** *Brain CT ordered or done* | | | YES | | | | | NO | | | NO DATA | |
|  | ***25.61*** *Structural abnormality detected by CT* | | | YES | | | | | NO | | | NO DATA | |
|  | ***25.7*** *Was there any referral to Neurology/or epilepsy specialty care?* | | | YES | | | | | NO | | | NO DATA | |
| **SEIZURE ASSESSMENT**  **Instructions for chart abstractor:** *If the reason for seizure is not specified as provoked then by default consider the seizure as unprovoked* | | | | | | | | | | | | | |
| 26 | Is there documentation of any seizure for this visit?  ***Instructions:***  *Only answer "YES" if pt reports the occurrence of seizure(s) at this visit* | | | YES | | | | | NO | | | NO DATA | |
|  | ***26.1*** *Is there any discussion of seizures since the last visit?*  *Instructions for abstractor- Answer “YES”, " if there is any documentation that physician inquired about whether any seizures occurred since the last visit, including no seizures since last visit)* | | | YES | | | | | NO | | | NO DATA | |
|  | ***26.2*** *Do the physician notes classify the documented seizures as “unprovoked”?* ***If yes, were any of the following classifications used to describe the seizure?*** | | | YES | | | | | NO | | | NO DATA | |
|  | *Generalized Types:* | | |  | | | | | | | | | |
|  | ***26.21*** *Petit mal (absence)* | | | YES | | | | | NO | | | NO DATA | |
|  | ***26.22*** *Grand mal (tonic-clonic, generalized convulsive)* | | | YES | | | | | NO | | | NO DATA | |
|  | ***26.23*** *Atonic* | | | YES | | | | | NO | | | NO DATA | |
|  | ***26.24*** *Myoclonic* | | | YES | | | | | NO | | | NO DATA | |
|  | ***26.25*** *Simple partial (w/ or w/o 2° generalized)* | | | YES | | | | | NO | | | NO DATA | |
|  | ***26.26*** *Complex partial (w/ or w/o 2° generalized)* | | | YES | | | | | NO | | | NO DATA | |
|  | ***26.27*** *Other* | | | Specify: Free text | | | | | | | | | |
|  | ***26.3*** *Do the physician notes indicate the etiology of the seizure?* ***If yes, was it due to any of the following items below*** | | | YES | | | | | NO | | | NO DATA | |
|  | ***26.31*** *Drug Addiction/ Withdrawal* | | | YES | | | | | NO | | | NO DATA | |
|  | ***26.311****Alcohol Addiction/Withdrawal* | | | YES | | | | | NO | | | NO DATA | |
|  | ***26.32*** *Renal Failure* | | | YES | | | | | NO | | | NO DATA | |
|  | ***26.33*** *Hepatic Failure* | | | YES | | | | | NO | | | NO DATA | |
|  | ***26.34*** *Poisoning/ Overdosage* | | | YES | | | | | NO | | | NO DATA | |
|  | ***26.35*** *Hypoglycemia (low-level of Glucose)* | | | YES | | | | | NO | | | NO DATA | |
|  | ***26.36*** *Hyponatremia ((low-level of Na)* | | | YES | | | | | NO | | | NO DATA | |
|  | ***26.37*** *Hypomagnesiemia ((low-level of Mg)* | | | YES | | | | | NO | | | NO DATA | |
|  | ***26.38*** *Shortly after CNS Infection/Insult* | | | YES | | | | | NO | | | NO DATA | |
|  | ***26.39*** *Shortly after Brain Trauma* | | | YES | | | | | NO | | | NO DATA | |
|  | ***26.40*** *Shortly after Stroke (CVA)* | | | YES | | | | | NO | | | NO DATA | |
|  | ***26.41*** *Other* | | | *Specify via free text* | | | | | | | | | |
| **PREVENTION/SCREENING/COUNSELING** | | | | | | | | | | | | | |
| 27 | Was any information concerning driving restrictions, safety and injury prevention provided to patient during this visit? | | | YES | | | | | NO | | NO DATA | | |
|  | 27.1 Was the patient educated about the impact of epilepsy and its treatment on lifestyle? | | | YES | | | | | NO | | NO DATA | | |
| 28 | Were any of the following patient assessment issues documented or discussed during this visit?(Instructions for abstractors- If Any of 28.1-28.5 is yes then 28 Yes) | | | YES | | | | | NO | | NO DATA | | |
|  | ***28.***1 E*ffects of epilepsy and its treatment on quality of life.*  Instructions for abstractor: If physician notes specifically mentions this | | | YES | | | | | NO | | NO DATA | | |
|  | ***28.2*** *Assessment of AED side-effects* | | | YES | | | | | NO | | NO DATA | | |
|  | ***28.3*** *Assessment of AED drug-drug interactions* | | | YES | | | | | NO | | NO DATA | | |
|  | ***28.4*** Assessment of AED impact *on bone health* | | | YES | | | | | NO | | NO DATA | | |
|  | ***28.5*** *Assessment of compliance to treatment and/or barriers to adherence to AED therapy* | | | YES | | | | | NO | | NO DATA | | |
| 29 | *Screening for Mood Disorder*  *Instructions for abstractor – look for assessment of anxiety, depression or mood disorder* | | | YES | | | | | NO | | NO DATA | | |
|  | - 1. *Diagnosis of mood disorder*   *Instructions for abstractor – look for diagnosis of anxiety, depression or a mood disorder* | | | YES | | | | | NO | | NO DATA | | |
| 30 | *Referral for mental health service* | | | YES | | | | | NO | | NO DATA | | |
| 31 | *Treatment for mood disorder*  *Instructions for abstractor- look for any drugs or psychosocial therapy for anxiety ,depression or mood disorder* | | | YES | | | | | NO | | NO DATA | | |
| **TREATMENT** | | | | | | | | | | | | | |
| 32 | Was the patient prescribed any new AEDs at this visit? | | | YES | | | | | NO | | NO DATA | | |
|  | ***32.1*** *At this visit, did the patient already have an AED prescription?* | | | YES | | | | | NO | | NO DATA | | |
|  | ***32.2*** *If 32.1 is Yes, was the AED taken as monotherapy?* | | | YES | | | | | NO | | NO DATA | | |
|  | ***32.3*** *If 32 and 32.1 were both answered as No or No Data, were any reasons for not prescribing documented?* | | | YES | | | | | NO | | NO DATA | | |
|  | **If 32 or 32.1 are Yes, answer 32.11- 32.18.** | | |  | | | | | | | | | |
|  | ***32.11-32.18:***  ***Instruction for abstractors: Specify the names of the AED that were discussed on this visit, and provide related information below.***  32.11 Please enter any AED drugs that the patient was taking, were newly prescribed, were discontinued, or had the dosage changed, at this visit  **Drug List**   | **Generic Name** | **Brand Name** | **Generic Name** | **Brand Name** | **Generic Name** | **Brand Name** | | --- | --- | --- | --- | --- | --- | | Tiagabine | Gabitril | Valproic Acid | Depakene | Phenytoin | Dilantin | | Phenobarbital | Luminol | Depacon | Fosphenytoin Sodium | Cerebyx | | Primidone | Mysoline | Depakote | Lamotrigine | Lamictal | | Clonazepam | Klonopin | Depakote ER | Ethosuximide | Zarontin | | Felbamate | Felbatol | Carbamazepine | Tegretol | Gabapentin | Neurontin | | Levetiracetam | Keppra | Tegretol XR | Topiramate | Topamax | | Keppra IV | Carbatrol | Oxacarbazepine | Trileptal | | Zonisamide | Zonegran | Epital | Pregabalin | Lyrica |      | **32.12 AED name** | **32.13 No Change** | **32.14 Newly Prescribed** | **32.15 Discontinued** | **32.16 Taper** | **32.17 Dose increase** | **32.18 Dose decrease** | | --- | --- | --- | --- | --- | --- | --- | |  |  |  |  |  |  |  | |  |  |  |  |  |  |  | |  |  |  |  |  |  |  | |  |  |  |  |  |  |  | | | | | | | | | | | | | |
|  | ***32.6*** *Enter AED names on this visit, with any additional information from physician notes*  *Instructions for abstractor – Copy paste AED related information directly from the physician notes. Include all available information related to drug dose, dose frequency, drug or dose changes, drugs added or removed, and side effects.* | | | | | | | | | | | | |
| **33** | **Is the patient taking or prescribed to take any of the following enzyme-inducing AEDs at this visit? Note. Typical Enzyme-inducing AEDs are marked with one asterisk (*)** | | | **YES** | | | | | **NO** | | **NO DATA** | | |
|  | **33.01** Phenobarbital (Luminol)* | | | YES | | | | | NO | | NO DATA | | |
|  | **33.02** Oxcarbazepine (Trileptal)**  *Limited enzyme induction- only to 2 enzymes; (effect at high doses or w/oral contraceptives)* | | | YES | | | | | NO | | NO DATA | | |
|  | **33.03** Primidone (Mysoline)* | | | YES | | | | | NO | | NO DATA | | |
|  | **33.04** Phenytoin (Dilantin)* | | | YES | | | | | NO | | NO DATA | | |
|  | **33.05** Valproic Acid (Depakote) | | | YES | | | | | NO | | NO DATA | | |
|  | **33.06** Lamotrigine (Lamictal) | | | YES | | | | | NO | | NO DATA | | |
|  | **33.07** Ethosuximide (Zarontin) | | | YES | | | | | NO | | NO DATA | | |
|  | **33.08** Gabapentin (Neurontin) | | | YES | | | | | NO | | NO DATA | | |
|  | **33.09** Topiramate (Topamax)**  *Enzyme inducing effect at high doses, only with OCP containing estrogen not progesterone* | | | YES | | | | | NO | | NO DATA | | |
|  | **33.10** Tiagabine (Gabitril) | | | YES | | | | | NO | | NO DATA | | |
|  | **33.11** Clonazepam (Klonopin) | | | YES | | | | | NO | | NO DATA | | |
|  | **33.12** Felbamate (Felbatol) | | | YES | | | | | NO | | NO DATA | | |
|  | **33.13** Levetiracetam (Keppra) | | | YES | | | | | NO | | NO DATA | | |
|  | **33.14** Zonisamide (Zonegran) | | | YES | | | | | NO | | NO DATA | | |
|  | **33.15** Pregabalin (Lyrica) | | | YES | | | | | NO | | NO DATA | | |
|  | **33.17** Carbamazepine(Tegretol)* | | | YES | | | | | NO | | NO DATA | | |
|  | **33.18** Valproate | | | YES | | | | | NO | | NO DATA | | |
| 34 | ***Is the patient taking any of the following medications? (risk of drug-drug interaction)*** | | | YES | | | | | NO | | NO DATA | | |
|  | ***34.1*** *Warfarin (Coumadin)* | | | *Drop-down menu* | | | | | NO | | NO DATA | | |
|  | ***34.2*** *Statins – except Provastatin* | | | *Drop-down menu* | | | | | | | | | |
|  | ***34.3*** *Anti-viral medications* | | | *Drop-down menu* | | | | | | | | | |
|  | ***34.4*** *Hormonal contraceptives* | | | *Drop-down menu* | | | | | | | | | |
|  | ***34.5*** *Antibiotics* | | | *Drop-down menu (Erythromycin, others)* | | | | | | | | | |
|  | ***34.6*** *Anti-Hypertensive’s*  *Note: if any of the 34.1-34.6 is YES then 34 Yes* | | | *Drop-down menu* | | | | | | | | | |
|  | ***34.6*** *Anti-Hypertensive’s*  *Note: if any of the 34.1-34.6 is YES then 34 Yes* | | | *Drop-down menu* | | | | | | | | | |
| **FEMALES** | | | | | | | | | | | | | |
| 39 | Does the medical record indicate that the physician discussed or carried out one or  more of the following: | | |  | | | | | | | | | |
|  | ***39.1*** *Counseling about Contraceptive change or dose adjustment if patient is on AED’s?* | | | YES | | | | | | | | | |
|  | ***39.2*** *Counseling about the impact of menopause on epilepsy?* | | | YES | | | | | NO | | NO DATA | | |
|  | ***39.3*** *Did the physician/nurse document that this patient is currently taking an oral contraceptive?*  ***Instructions:***  *Only answer "YES" if it clearly states pt is taking OCP whereas answering "NO" means physician documented no OCP is currently being taken by patient* | | | YES | | | | | NO | | NO DATA | | |
|  | ***39.31*** *If yes, is there any indication in the physician notes/drug list that this woman is also taking enzyme inducing AED’s? Refer to the list below:*   - Carbamazepine (Tegretol, Tegretol XR, Carbatrol, Epital) - Phenobarbital (Luminol) - Oxacarbazepine (Trileptal) - Primidone (Mysoline) - Phenytoin (Dilantin) | | | YES | | | | | NO | | NO DATA | | |
|  | ***39.32*** *If yes, is there any indication in the chart to address the decreased effectiveness of the oral contraceptive or Lamotrigine* | | | YES | | | | | NO | | NO DATA | | |
|  | ***39.33*** *Any change of oral contraceptive* | | | YES | | | | | NO | | NO DATA | | |
|  | ***39.4*** *Counseling about the impact of pregnancy on epilepsy?* | | | YES | | | | | NO | | NO DATA | | |
|  | ***39.41*** *Is there any documentation that the woman is on supplemental Folate, at least 400ucg*  *Instruction for chart abstractor: Answer yes also if a patient is on Multivitamins, which includes supplemental Folate, at least 400ucg* | | | YES | | | | | NO | | NO DATA | | |
|  | ***39.5*** *Is there any documentation that patient is pregnant?* | | | YES | | | | | NO | | NO DATA | | |
|  | ***39.51 If yes****, is there any reference that a neurologist/epileptologist AND an obstetrician- gynecologist are co-managing the patient?* | | | YES | | | | | NO | | NO DATA | | |
|  | **LAB INVESTIGATIONS**  **Instructions for chart abstractor**  **Items40-41:** *click the “Chart” tab (located on the top most rows, second from left) then “Documents” (5^th^ row, far right) then “labs” also view Physician/RN’s.* | | |  | | | | | NO | | NO DATA | | |
|  | Were AED blood Levels ordered/completed  Instructions for abstractor- Consider “YES” if order for AED level(s) or result(s) are documented at this visit | | | YES | | | | | | | | | |
| 40 | *Were bone health investigations ordered/ completed?* | | | YES | | | | | NO/ NO DATA | | | | |
| 41 | - 1. *Calcium levels*   *Instruction: Select “Yes” only if the chart specifically mentions that Ca levels were ordered or completed. Do not make assumptions since Q.42 addresses any captured for blood chemistry.* | | | YES | | | | | NO/ NO DATA | | | | |
|  | ***41.2*** *25, Hydroxyl Vitamin D levels* | | | YES | | | | | NO/ NO DATA | | | | |
|  | ***41.3*** *Parathyroid hormone levels* | | | YES | | | | | NO/ NO DATA | | | | |
|  | ***41.4*** *Dexa Scan/Bone Scan* | | | YES | | | | | NO/ NO DATA | | | | |
|  | **Were blood chemistries (including B-met, C-met and Panel 9) ordered / completed?** | | | YES | | | | | NO/ NO DATA | | | | |
| 42 | **Enter all drugs the patient was taking at this visit (copy from physician notes or check None)** | | |  | | | | | NO/ NO DATA | | | | |
| **50** | **COMORBIDITIES** | | |  | | | | | | | | | |
|  | Enter the comorbidities that the patient has at the time of the interview:  *Instruction: look at the most recent physician note prior to phone interview, where comorbidities are listed.* | | |  | | | | | | | | | |
| **51** | 51.1 Date of the most recent visit before the phone interview date, where comorbidities are listed | | |  | | | | | | | | | |
|  | 51.2 Physical comorbidities | | | Check box for the main diseases of interest | | | | | | | | | |
|  | 51.3 Mental comorbidities | | | Check box for the main diseases of interest | | | | | | | | | |
|  | 51.4 All comorbidities | | | Copy/ paste all | | | | | | | | | |
| **52** | Does the patient report side effects? | | | YES | | | NO SIDE EFFECTS | | | | | | NO DATA |
|  | 52.1 Specify side effects | | | Free text | | | | | | | | | |
|  | 52.2 Does the physician attribute side-effects to AED(s)? | | YES | | NO | PHYSICIAN UNSURE | | | | | | | NO DATA |
|  | 52.3 Specify AED(s) | | | Enter up to 3 AEDs | | | | | | | | | |
